# Relationship between volume and outcome for congenital diaphragmatic hernia: a systematic review protocol

**DOI:** 10.1186/s13643-018-0872-9

**Published:** 2018-11-13

**Authors:** Johannes Morche, Tim Mathes, Anja Jacobs, Barbara Pietsch, Lucas Wessel, Sabine Gruber, Edmund A. M. Neugebauer, Dawid Pieper

**Affiliations:** 1 Institute for Research in Operative Medicine, Faculty of Health, School of Medicine, Witten/Herdecke University, Ostmerheimer Str. 200, Building 38, 51109 Cologne, Germany; 2 Medical Consultancy Department, Federal Joint Committee, Wegelystraße 8, 10623 Berlin, Germany; 3 Department of Pediatric Surgery, Medical Faculty Mannheim (UMM), University of Heidelberg, Theodor-Kutzer-Ufer 1-3, 68167 Mannheim, Germany; 4 Faculty of Medicine, Brandenburg Medical School Theodor Fontane, Campus Neuruppin, Fehrbelliner Straße 38, 16816 Neuruppin, Germany

**Keywords:** Congenital diaphragmatic hernia, Congenital abnormalities, Hospitals, High-Volume, Hospitals, Low-Volume, Hospital volume, Surgeon volume, Volume–outcome

## Abstract

**Background:**

Congenital diaphragmatic hernia is a rare and life-threatening anomaly that occurs during fetal development and results in an incomplete or incorrect formation of the diaphragm. Surgical therapy of the diaphragm should be performed after clinical stabilization of the neonate. Higher hospital or surgeon volume has previously been found to be associated with better clinical outcomes for different especially high-risk, low-volume procedures. Therefore, we aim to examine the relationship between hospital or surgeon volume and outcomes for congenital diaphragmatic hernia.

**Methods:**

This systematic review protocol has been designed according to the Preferred Reporting Items for Systematic Reviews and Meta-Analysis Protocol. We will perform a systematic literature search in MEDLINE, Embase, CINAHL and Biosis Previews without applying any limitations. In addition, we will search for relevant conference abstracts. We will screen titles and abstracts of retrieved studies, obtain potentially relevant full texts, and assess the eligibility of those full texts against our inclusion criteria. We will include comparative studies analyzing the relationship between hospital or surgeon volume and clinical outcomes. We will systematically assess risk of bias of included studies and extract data on the study design, patient characteristics, case-mix adjustments, statistical methods, hospital and surgeon volume, and outcomes into standardized tables. Title and abstract screening, full-text screening, critical appraisal, and data extraction of results will be conducted by two reviewers independently. Other data will be extracted by one reviewer and checked for accuracy by a second one. Any disagreements will be resolved by discussion. We will not perform a meta-analysis as we expect included studies to be clinically and methodologically very diverse. We will synthesize findings from primary studies in a structured narrative way and using GRADE.

**Discussion:**

Given the lack of a comprehensive summary of findings on the relationship between hospital or surgeon volume and outcomes for congenital diaphragmatic hernia, this systematic review will put things right. Results can be used to inform decision makers or clinicians and to adapt medical care.

**Systematic review registration:**

PROSPERO (CRD42018090231)

**Electronic supplementary material:**

The online version of this article (10.1186/s13643-018-0872-9) contains supplementary material, which is available to authorized users.

## Background

In surgical disciplines, lots of studies have been published on the volume–outcome relationship starting with Luft et al. in the late 1970s [1, 2]. Many systematic reviews indicate a positive relationship between hospital as well as surgeon volume and clinical outcomes for different surgical procedures [3–6]. This relationship seems to be stronger for high-risk, low-volume surgeries [7–10].

This characterization —high risk and low volume— applies to surgery for congenital diaphragmatic hernia (CDH) with an incidence of 1 per 2500–5000 births [11–14] and survival rates of 30 to 85% based on population studies [15–18] and depending among others on comorbidities. CDH is an anomaly that occurs in the early stages of fetal development and results in an incomplete or incorrect formation of the diaphragm. Consequently, abdominal organs might move upward in the thoracic cavity leading to abnormal lung development and function. Lung hypoplasia of the ipsi- and contralateral side, depending on the size of the defect and pulmonary hypertension, are the main problems leading to mortality and morbidity. As hypothesis, the dual-hit, combining primary hypoplasia and secondary suppression, is favorized [19]. CDH is commonly classified according to the anatomic location of the defect — i.e., a posterolateral defect (Bochdalek hernia), an anterior defect (Morgagni hernia), or other defects, including central septum transversum-type, total absence of diaphragm, or esophageal hiatal hernia [20]. According to a classification of the Congenital Diaphragmatic Hernia Study Group, the severity might be based on the size and the characteristics of the defect: A — small defects entirely surrounded by muscle; B — defects with < 50% of chest wall devoid of diaphragm tissue; C — defects with > 50% of chest wall devoid of diaphragm tissue; and D — complete or near complete absence of the diaphragm [21].

CDH can be diagnosed prenatally or postnatally. In 50 to 85% of cases, CDH is diagnosed prenatally by fetal imaging when abdominal contents, e.g., bowel or stomach, moved upwards in the thoracic cavity [20, 22]. Postnatally, CDH can be diagnosed by X-ray when abdominal contents are present in the thoracic cavity [22, 23].

The CDH EURO Consortium recommends that surgical therapy for CDH should be performed after clinical stabilization of the neonate. It defines clinical stabilization as a normal mean arterial blood pressure according to the gestational age, preductal saturation levels of 85 to 95% on fractional inspired oxygen below 50%, lactate below 3 mmol/l, and a urine output of more than 1 ml/kg/h [24]. Additionally, it states that it is unclear whether neonates with CDH benefit from extracorporeal membrane oxygenation (ECMO). It recommends that surgery can be performed while the neonate is on ECMO [24]. Surgical therapy for CDH includes minimally invasive and open procedures. Minimally invasive surgery which includes laparoscopy and thoracoscopy might be considered for neonates with mild disease. For neonates with Morgagni’s hernia, laparoscopy is preferred to thoracoscopy [20, 23]. Open surgery including laparotomy and thoracotomy needs to be conducted on cases with large or complicated defects or if neonates require ECMO. Laparotomy seems to be used more frequently than thoracotomy [20].

To the best of our knowledge, there is only one systematic review published in 2013 and considering evidence up to April 2012 that includes primary studies on the relationship between volume and outcomes in neonates with diaphragmatic hernia [25]. Three out of four included primary studies demonstrated a positive association between hospital volume and mortality whereas one study showed a negative association. The CDH EURO Consortium states that the effect of hospital volume on mortality is unclear [24] by referring to two studies on the relationship between volume and outcomes [26, 27]. The mentioned systematic review did not focus on neonates with diaphragmatic hernia so that results on this condition were presented only briefly for hospital volume and lack for surgeon volume [25]. Additionally, approximately half of the reviews are out of date after 5.5 years, though it must be acknowledged that this estimate stems from systematic reviews of randomized controlled trials and might for that reason not necessarily hold true for systematic reviews of observational studies [28]. Hence, it seems reasonable to conduct a systematic review on this issue. It is suggested that outcomes (especially mortality) of neonates suffering from CDH that are operated in a high-volume hospital or by a high-volume surgeon are favorable compared to outcomes of neonates that are operated in lower volume hospitals or by lower volume surgeons. The aim of our systematic review is to examine the available literature on the relationship between hospital as well as surgeon volume and outcomes for CDH.

## Methods/design

The protocol follows the Preferred Reporting Items for Systematic Reviews and Meta-Analyses Protocol (see Additional file 1) [29]. The PROSPERO (http://www.crd.york.ac.uk/PROSPERO) registration number is CRD42018090231.

### Literature search strategy

We will perform a systematic literature search to identify all published studies on the relationship between hospital or surgeon volume and clinical outcomes for CDH. MEDLINE (via PubMed), Embase (via Ovid), CINAHL (via EBSCO), and Biosis Previews (via Ovid) will be searched from inception until the day of search. We will use a combination of Medical Subject Headings (MeSH) and free text words (all search strategies can be found in Additional file 2). No language restrictions or other limits will be applied. Reference lists of relevant articles will be inspected to identify additional articles that could have been missed by the search strategy. Additionally, we will screen individual conference proceedings (see Additional file 3 for a list of conferences). Furthermore, we will use search engines such as google scholar to identify gray literature (see Additional file 2 for all searched engines). We will contact authors for detailed information in case of perceived relevance of abstracts. The search results will be uploaded and managed using Endnote.

### Eligibility criteria

The following inclusion criteria will be applied to each publication: the subject of the study is congenital diaphragmatic hernia; the study has a comparative design, we expect to include mostly observational studies; however, if available, we will also include subgroup analyses of experimental study designs (e.g., RCTs); clinical outcomes (e.g., mortality, morbidity) are studied (see “Outcomes and priorization”); volume is assessed as a categorical variable or a continuous variable; the study describes more than one hospital or surgeon; and the publication is written in English or German.

### Study selection

All titles and abstracts of articles identified through systematic literature search will be screened independently by two members of the research team. The full texts of potentially eligible articles will be obtained, and the eligibility of the full texts against the review inclusion criteria will be assessed by two reviewers independently. Any disagreements will be resolved by discussion. The study selection will be documented in Endnote.

### Data collection

For each included publication, the following characteristics will be extracted: year of publication, country, study design and methodology, data source, study period, definition of CDH, number of patients, number of hospitals and/or surgeons, patient characteristics, case-mix adjustments, statistical methods, volume categories for hospitals, volume categories for surgeons, analyzed outcomes, results regarding these outcomes, and the funding source as well as authors’ reported conflicts of interest. All data will be extracted into structured, beforehand piloted summary tables using Microsoft Word. Results will be extracted independently by two reviewers. Other data will be extracted by one reviewer and checked for accuracy by a second reviewer who will read the paper in detail and ensure that no relevant information was missed. Any disagreements will be discussed until consensus is reached.

Study results will be recorded separately for each unit (surgeon or hospital) and outcome. If study authors present adjusted and unadjusted results, we will focus our synthesis on adjusted results. Nevertheless, we will extract unadjusted results as well. In case volume is classified in categories, we will report hazard ratios for time-to-event analyses and odds ratios or relative risks for dichotomous outcomes and mean difference for continuous outcomes. We will provide the measures with 95% confidence levels if reported by authors or calculable given the available data. Moreover, we will recalculate effect measures (e.g., odds ratio, hazard ratio, relative risk) so that higher volume is compared to lower volume and not vice versa. We will calculate effect measures if results can be calculated from information in the text, but effect measures are not presented. In case volume is treated as a continuous variable, we will present results of the analyses conducted within primary studies. We will contact study authors for clarification in any case of uncertainty regarding data collection and critical appraisal.

### Outcomes and prioritization

The primary outcomes that will be analyzed in our systematic review are survival and mortality (surgery-related; up to discharge; long-term, e.g., 2-year or 5-year) given that CDH is a life-threatening disease. Secondary outcomes are recurrence of hernia, bleeding complication/major hemorrhage, chronic lung disease (e.g., dependence of oxygen after day 56 postpartum), failure to thrive, skeletal abnormalities (e.g., chest asymmetry, pectus deformities, vertebral anomalies such as kyphosis and sclerosis), gastroesophageal reflux disease, hypercapnia, acidosis, patch repair, small bowel obstruction, days of ventilation, and length of stay as these were reported as important outcomes in previous studies on CDH, e.g., when comparing different surgical techniques [20, 30].

### Critical appraisal

So far, there is no consensus on which tool to use for quality appraisal of studies on the relationship between volume and outcomes when conducting a systematic review [7]. These studies are almost exclusively based on observational data. We will use the tool for assessing risk of bias in non-randomized studies of interventions (ROBINS-I) that was recently developed by members of Cochrane methods groups [31]. Although being primarily designed for assessment of non-randomized studies of interventions, it might also be used to evaluate observational studies where the intervention is an exposure (i.e., risk factor — low volume). In case an already announced version of the tool especially modified for studies of exposures (ROBINS-E) will be published [32, 33], we will use this version if it can be applied to volume–outcome studies. Methodological quality of the eligible studies will be assessed independently by two reviewers. Any disagreements will be resolved by discussion.

### Data synthesis

We expect the included studies to be clinically and methodologically diverse, e.g., including neonates with diverse illness severities, using different methods of testing and applying different types of operative repair [20] as well as using different cutoff values for high and low volume [7, 8]. Therefore, we will provide a systematic narrative synthesis to summarize and explain the findings of the included studies. The quality of the body of evidence will be assessed by using GRADEpro GDT. We will synthesize findings based on outcomes separately for each unit (surgeon or hospital) and consider risk of bias, imprecision, indirectness, inconsistency, and publication bias as well as the magnitude of treatment (or exposure) effect, the presence of a dose–response gradient, and plausible residual confounding as recommended by GRADE [34]. We are aware that the risk of publication bias is particularly high for studies based on automatically collected observational data (e.g., in electronic medical records or registries) [35]. However, to our knowledge, there is no tool to assess presence and extent of publication bias when results are not pooled across studies. Therefore, we will discuss potential impact of publication bias narratively. In this context, we will also consider the linguistic limitation of our systematic review of including solely documents written in English or German.

The systematic review will be conducted according to the Preferred Reporting Items for Systematic Reviews and Meta-analyses (PRISMA) [36].

## Discussion

The aim of this review is to evaluate the relationship between hospital or surgeon volume and outcomes for surgeries on CDH. Despite an existing minimum volume standard on CDH in the Netherlands [37], there is no up-to-date analysis that synthesizes results from different studies on the volume–outcome relationship systematically and comprehensively. Therefore, it is important to evaluate this relationship so that insights can be used to inform decision makers or clinicians and to adapt medical care.

## Additional files

Additional file 1: PRISMA-P 2015 checklist. (DOCX 30 kb)

Additional file 2: Search strategies for medical databases and search engines. (DOCX 19 kb)

Additional file 3: List of conferences. (DOCX 14 kb)

## Abbreviations

CDH: Congenital diaphragmatic hernia
GRADE: The Grading of Recommendations Assessment, Development and Evaluation
RCT: Randomized controlled trial
ROBINS-E: Risk of bias in non-randomized studies of exposures
ROBINS-I: Risk of bias in non-randomized studies of intervention
