# Supplementary material for: Relationship between volume and outcome for congenital diaphragmatic hernia: a systematic review protocol
Source: Syst Rev. 2018 Nov 13;7:185. doi: 10.1186/s13643-018-0872-9 (PMC6234699; doi:10.1186/s13643-018-0872-9)
Supplement: Supplementary file 2 — Search strategies for medical databases and search engines. (DOCX 19 kb) [file 13643_2018_872_MOESM2_ESM.docx]

**Additional file 2: Search strategies for medical databases and search engines**

Medical databases

Medline (Pubmed)

| # | #Suchfrage |
| --- | --- |
| #1 | "hernias, diaphragmatic, congenital"[mh] |
| #2 | ((congenital[tiab] AND diaphragm*[tiab] AND (hernia*[tiab] OR defect*[tiab]))) |
| #3 | ((Bochdalek[tiab] OR morgagni*[tiab]) AND (hernia*[tiab])) |
| #4 | (#1 OR #2 OR #3) |
| #5 | "hospitals, low volume"[mh] |
| #6 | „hospitals, high-volume[mh] |
| #7 | "workload"[mh] |
| #8 | ("hospitals/statistics and numerical data"[mh]) |
| #9 | ("hospitals, pediatric/statistics and numerical data"[mh]) |
| #10 | ("intensive care units, pediatric/statistics and numerical data"[mh]) |
| #11 | ("intensive care, neonatal/statistics and numerical data"[mh]) |
| #12 | volume*[tiab] |
| #13 | (regionali*[tiab] OR centrali*[tiab]) |
| #14 | (workload*[tiab] OR caseload*[tiab]) |
| #15 | ((#5 OR #6 OR #7 OR #8 OR #9 OR #10 OR #11 OR #12 OR #13 OR #14)) |
| #16 | "outcome assessment health care"[mh] |
| #17 | Mortality[mh] |
| #18 | ((outcom*[tiab] OR mortality[tiab] OR surviv*[tiab])) |
| #19 | (#16 OR #17 OR #18) |
| #20 | (#4 AND #15 AND #19) |
| #21 | "Hernias, Diaphragmatic, Congenital/statistics and numerical data"[mh] |
| #22 | (#20 OR #21) |

Embase (OVID)

| # | Suchfrage |
| --- | --- |
| 1 | exp congenital diaphragm hernia/ |
| 2 | (congenital adj3 diaphragm* adj3 (hernia* or defect*)).ab,ot,ti. |
| 3 | ((Bochdalek or morgagni*) and hernia*).ab,ot,ti. |
| 4 | 1 or 2 or 3 |
| 5 | exp high volume hospital/ |
| 6 | exp low volume hospital/ |
| 7 | *hospital/ |
| 8 | *newborn intensive care/ |
| 9 | exp workload/ |
| 10 | (workload* or caseload*).ab,ot,ti. |
| 11 | (regionali* or centrali*).ab,ot,ti |
| 12 | (volume*).ab,ot,ti. |
| 13 | 5 or 6 or 7 or 8 or 9 or 10 or 11 or 12 |
| 14 | exp outcome assessment/ or exp treatment outcome/ |
| 15 | exp hospital mortality/ |
| 16 | exp infant mortality/ |
| 17 | exp newborn mortality/ |
| 18 | exp survival rate/ |
| 19 | (outcom* or mortality or surviv*).ab,ot,ti. |
| 20 | 14 or 15 or 16 or 17 or 18 or 19 |
| 21 | 4 and 13 and 20 |

CINAHL (EBSCO)

| # | Suchfrage |
| --- | --- |
| 1 | (MH "Hernia, Diaphragmatic, Congenital") |
| 2 | TI congenital diaphragm* hernia* |
| 3 | AB congenital diaphragm* hernia* |
| 4 | TX Bochdalek AND TX hernia* |
| 5 | TX morgagni AND TX hernia* |
| 6 | S1 OR S2 OR S3 OR S4 OR S5 |
| 7 | TX volume* OR TX workload* OR TX caseload* OR TX regionali* OR TX centrali* |
| 8 | TX outcom* OR TX mortality OR TX surviv* |
| 9 | S6 AND S7 AND S8 |
| 10 | S6 AND S7 AND S8 Limiters - Exclude MEDLINE records |

Biosis Previews (OVID)

| # | Suchfrage |
| --- | --- |
| 1 | (congenital and diaphragm* and (hernia* or defect*)).af. |
| 2 | ((Bochdalek or morgagni) and hernia*).af. |
| 3 | (volume* or workload* or caseload* or regionali* or centrali*).af. |
| 4 | (outcom* or mortality or surviv*).af. |
| 5 | 1 or 2 |
| 6 | 3 and 4 and 5 |
| 7 | limit 6 to human |

Search engines

We will use different spellings, variations and combinations of the following terms to identify grey literature in google scholar (https://scholar.google.de/), base-search (<https://www.base-search.net/Search/Advanced>), LIVIVO (<https://www.livivo.de/>), Karlsruher Virtueller Katalog (<https://kvk.bibliothek.kit.edu/>), and Deutsche Nationalbibliothek (<https://portal.dnb.de/opac.htm>):

congenital diaphragmatic hernia, Bochdalek, Morgagni, hospital, volume, outcome, mortality, survival; angeborene Zwerchfellhernie, Krankenhaus, Chirurgie, Operativer Eingriff
