# Supplementary material for: Relationship between volume and outcome for congenital diaphragmatic hernia: a systematic review protocol
Source: Syst Rev. 2018 Nov 13;7:185. doi: 10.1186/s13643-018-0872-9 (PMC6234699; doi:10.1186/s13643-018-0872-9)
Supplement: Supplementary file 3 — List of conferences. (DOCX 14 kb) [file 13643_2018_872_MOESM3_ESM.docx]

**Additional file 3: List of conferences**

List of conferences:

American Congress on Pediatric Surgery (APSA)

Annual Congress on Neonatology & Pediatrics

Annual World Congress on Neonatology

Annual World Congress on Pediatrics

British Congress of Pediatric Surgery (BAPS)

Congenital Diaphragmatic Hernia International Workshop Symposium / Congenital Diaphragmatic Hernia Workshop

European Congress of Pediatric Surgery (EUPSA)

European Pediatrics Conference

European Pediatrics Congress

Global Pediatrics

Global Summit on Pediatrics, Neonatology & Primary Care

International CDH Conference

International Conference on Maternal Fetal Neonatal Medicine

International Conference on Neonatology and perinatology

International Conference on Pediatrics and Pediatric Surgery (EUPSA, WOFAPS)

World Congress on Clinical Pediatrics

World Congress on Neonatology & Perinatology

World Congress on Pediatrics & Neonatal Care

World Neonatology Meeting

World Pediatric Congress

World Pediatrics Conference
